# Targeting Ferroptosis in Sensorineural Hearing Loss: A Mechanistic Review of Therapeutic Opportunities

**DOI:** 10.3390/cimb47110876

**Published:** 2025-10-22

**Authors:** Han Liu, Xinlei Chu, Meiqi Liao, Jie Wang, Hongbing Zhang, Lei Han

**Affiliations:** 1 Department of Occupational Disease Prevention, Jiangsu Provincial Center for Disease Control and Prevention (Jiangsu Provincial Academy of Preventive Medicine), Nanjing 210000, China; 2 School of Public Health, Nanjing Medical University, Nanjing 211166, China; 3 School of Public Health, Southeast University, Nanjing 210009, China

**Keywords:** ferroptosis, sensorineural hearing loss, therapeutic target, otoprotection, drug discovery, oxidative stress, hair cell preservation

## Abstract

Ferroptosis, an iron-dependent form of regulated cell death, is emerging as a critical pathogenic mechanism and a highly promising therapeutic target in sensorineural hearing loss (SNHL). The irreversible loss of auditory hair cells, the hallmark of SNHL, creates an urgent need for novel therapeutic strategies. This review provides a translational perspective on ferroptosis, connecting its core molecular machinery to tangible opportunities for otoprotection. We systematically analyze three key targetable nodes: the iron metabolic pathways that fuel the process; the lipid peroxidation machinery that executes membrane damage; and the collapse of the System Xc^−^–GSH–GPX4 antioxidant axis. By framing the disease mechanism through these actionable targets, we highlight a clear rationale for developing new hearing preservation therapies. We conclude by surveying the most promising pharmacological approaches, including iron chelators, radical-trapping antioxidants, and bioactive natural products, offering a strategic roadmap for future drug discovery in audiology.

## 1. Introduction

Hearing loss is a major global public health challenge, with environmental and age-related factors contributing to the prevalence of hearing loss [1]. There are two primary forms of hearing loss: conductive hearing loss and sensorineural hearing loss (SNHL). Conductive hearing loss is a mechanical impairment caused by problems in the outer or middle ear that reduce the efficient transmission of sound to the inner ear. SNHL arises from damage to or dysfunction of structures within the inner ear, such as the sensory hair cells, their synapses with the auditory nerve, or the stria vascularis. It can also result from damage to the auditory nerve pathways themselves. Common causes include aging (presbycusis), prolonged exposure to loud noise, ototoxic medications, and genetic factors. SNHL is the most common type of permanent hearing loss. Noise exposure is the best-known environmental factor contributing to hearing loss, with high-intensity noise causing the death of cochlear hair cells (both inner and outer hair cells) [2,3]. The permanent loss of cochlear hair cells, which are incapable of regeneration in mammals, is the definitive pathological hallmark of sensorineural hearing loss (SNHL). For decades, the demise of these cells has been primarily attributed to apoptosis and necrosis. Central to these cell death pathways is the pivotal role of oxidative stress, an imbalance between the excessive production of reactive oxygen species (ROS) and the cochlea’s intrinsic antioxidant defenses. This oxidative onslaught triggers a cascade of cellular damage, including mitochondrial dysfunction and the activation of apoptotic signaling, ultimately leading to irreversible hearing impairment [4,5].

Recently, ferroptosis, a novel form of regulated cell death (RCD), has emerged as a critical player in the pathologies associated with oxidative stress. Ferroptosis is uniquely characterized by the iron-dependent accumulation of lipid peroxides, culminating in cell membrane rupture. Its execution is tightly linked to factors such as intracellular iron overload, depletion of glutathione peroxidase 4 (GPX4), and the peroxidation of polyunsaturated fatty acids (PUFAs). Intracellular free iron (Fe^2+^) catalyzes lipid peroxidation through the Fenton reaction, initiating a chain reaction of free radicals. Processes such as ferritinophagy further exacerbate the expansion of the unstable intracellular iron pool [6]. The canonical defense against ferroptosis is orchestrated by the System Xc^−^–glutathione (GSH)–glutathione peroxidase 4 (GPX4) axis [7]. System Xc^−^ mediates cystine uptake for glutathione (GSH) synthesis, while GPX4 utilizes GSH to reduce phospholipid hydroperoxides to non-toxic alcohols, thereby terminating the lipid peroxidation cascade. Disruption at any node of this axis can render cells susceptible to ferroptosis. Acyl-CoA synthetase long-chain family member 4 (ACSL4) and LPCAT3 facilitate the incorporation of polyunsaturated fatty acids (PUFAs) into membrane phospholipids, generating peroxidation-sensitive substrates that fuel ferroptosis [8]. Additionally, GPX4-independent pathways, including the FSP1/CoQ10 and GCH1/BH4 systems, can also inhibit lipid peroxidation [9,10,11].

The intrinsic characteristics of cochlear hair cells—high metabolic activity, abundant polyunsaturated fatty acids (PUFAs), and susceptibility to oxidative damage—create a microenvironment highly conducive to ferroptosis. Ferroptosis thus likely represents a significant and previously overlooked pathway in hair cell injury. Although ferroptosis has been extensively studied in a variety of areas including diabetes [5], tumors [12], Parkinson’s disease [13], atherosclerosis [14], and ischemia–reperfusion injury [15], it remains relatively underexplored in auditory patho-physiology. In this review, we aim to provide an in-depth look at the molecular mechanisms of ferroptosis and its role in the pathogenesis of SNHL. We will systematically discuss the evidence linking ferroptosis to different forms of SNHL and explore the key regulatory pathways involved, highlighting the potential therapeutic strategies that target ferroptosis to prevent or treat hearing loss.

## 2. Ferroptosis and Sensorineural Hearing Loss

The mammalian cochlea contains two distinct populations of mechanosensory receptors, inner hair cells (IHCs) and outer hair cells (OHCs), which are distinguished by their unique morphology and function. IHCs are the primary afferent transducers, converting mechanical stimuli into neural signals that are transmitted to the central nervous system. Conversely, OHCs act as biological amplifiers, modulating cochlear mechanics to enhance auditory sensitivity and frequency selectivity [16,17,18]. Both IHCs and OHCs perform mechanoelectrical transduction via their apical stereocilia bundles, which is essential for the initial encoding of sound.

SNHL is a form of hearing impairment arising from pathology within the inner ear, auditory nerve, or central auditory pathways. The core pathophysiology involves deficits in the transduction, transmission, or processing of acoustic signals. While early audiological diagnostics may not precisely distinguish cochlear from retrocochlear lesions, the vast majority of SNHL cases originate within the cochlea itself. As the primary sensory organ of the inner ear, the cochlea is responsible for converting mechanical sound vibrations into electrical neural impulses [19]. Cochlear function is critically dependent on tightly regulated ion transport, which establishes the unique electrochemical gradients essential for hearing. Consequently, disruptions to this delicate homeostasis, whether genetic or acquired, can cause metabolic imbalances and subsequent hearing impairment. The cochlear blood supply, maintained by the stria vascularis, is highly vulnerable to insults like noise exposure, ototoxic agents, and systemic vascular compromise [20]. Dysfunction of the stria vascularis compromises cochlear ionic homeostasis, leading to fluid-space imbalances and functional decline.

As an organ with high metabolic activity and oxygen demand, the cochlea is exceptionally vulnerable to a range of pathological insults. The pathogenesis of hearing loss involves multi-level mechanisms, principally direct mechanical trauma and subsequent biochemical and metabolic dysregulation. High-intensity noise exposure, a major environmental cause of hearing damage, operates through a dual mechanism. First, intense sound pressure directly induces mechanical damage, such as the shearing and fracture of hair cell stereocilia bundles, disruption of the reticular lamina, and compromising of intercellular junctions. This form of mechanical damage is typically acute and permanent. Second, noise exposure initiates a cascade of secondary biochemical events, including compromised cochlear blood flow, glutamate-mediated excitotoxicity, and, most critically, overwhelming oxidative stress [4,21].

Within the spectrum of biochemical insults, oxidative stress represents a convergent pathway for various etiologies of hearing loss. Cochlear degeneration induced by noise, ototoxic drugs, and aging is consistently linked to the excessive accumulation of reactive oxygen species (ROS) [4]. The cardinal features of ferroptosis—namely, intracellular iron overload, collapse of the GPX4-mediated antioxidant system, and massive lipid peroxide accumulation—strongly correlate with the molecular pathology observed following cochlear injury. The high concentration of polyunsaturated fatty acids in the membranes of hair cells and spiral ganglion neurons renders them particularly susceptible to iron-dependent oxidative damage. For instance, in superficial siderosis, chronic subarachnoid hemorrhage causes hemosiderin deposition along auditory neural pathways, directly inducing iron-dependent neurodegeneration [22]. Similarly, HFE gene mutations in hereditary hemochromatosis lead to systemic iron overload, which may indirectly damage auditory neurons by compromising the blood-labyrinth barrier [23]. In complex syndromes involving hemochromatosis, Wilson’s disease, and superficial siderosis, synergistic dysregulation of iron and copper metabolism can exacerbate oxidative stress, thereby promoting ferroptosis [24]. Collectively, preclinical and clinical evidence delineates a pathogenic cascade: dysregulated iron homeostasis within the auditory system triggers oxidative stress, which in turn drives ferroptosis, culminating in auditory dysfunction. Importantly, mechanical trauma and biochemical insults like ferroptosis are not mutually exclusive but often interact synergistically, creating a vicious cycle that accelerates the progression of hearing loss.

### 2.1. The Role of Ferroptosis in Age-Related Hearing Loss

Age-related hearing loss (ARHL) is the most prevalent sensory deficit among the elderly population. It is characterized as a progressive, bilateral, and symmetric sensorineural hearing loss, most prominently affecting higher frequencies. The etiology of ARHL is multifactorial, involving a combination of genetic predisposition, cumulative environmental insults, and the physiological effects of aging. Pathological investigations have traditionally focused on microscopic cochlear alterations, including transcriptional dysregulation in hair cells, exemplified by the downregulation of key genes like *Slc26a5* [25]. Mitochondrial dysfunction is also a key feature, characterized by cristae disruption, reduced expression of cytochrome c oxidase subunits, and ROS accumulation. These changes promote a chronic inflammatory state with elevated pro-inflammatory cytokines (e.g., IL-1β, IL-6, TNF-α), which in turn activates multiple cell death pathways, including apoptosis and ferroptosis [26]. At the molecular level, these cell death programs involve regulators such as p53 in early apoptosis and Bcl-2 in both apoptosis and necrosis [27]. These cascading effects ultimately lead to the degeneration of key auditory structures, including outer and inner hair cells, spiral ganglion neurons, and the stria vascularis [25,28]. Furthermore, the link between systemic health and hearing is underscored by studies showing that metabolic disorders, such as type 2 diabetes, can exacerbate the progression of ARHL [29].

Emerging evidence implicates ferroptosis, an iron-dependent form of regulated cell death driven by lipid peroxidation, as a key pathogenic mechanism in ARHL. Within the aging cochlea, chronic oxidative stress and mitochondrial dysfunction create a cellular milieu that sensitizes auditory cells to ferroptosis. Consistent with this, aging auditory tissues exhibit iron accumulation and mitochondrial abnormalities characteristic of ferroptosis, including reduced mitochondrial volume, increased membrane density, and cristae effacement [26,30,31]. These findings position ferroptosis as a tractable therapeutic target for ARHL. For instance, the ferroptosis inhibitor CMS121 ameliorates oxidative stress and lipid peroxidation, thereby improving auditory function and preserving ribbon synapses in the SAMP8 mouse model of aging [32]. Directly targeting iron overload, a core component of ferroptosis, has also shown promise. In a mouse model of ARHL, the iron chelator deferoxamine (DFO) reduced iron-induced damage in central auditory neurons, suggesting a potential intervention for the central components of presbycusis [33].

Across the entire auditory pathway, from cochlear hair cells to cortical neurons, age-related oxidative stress, mitochondrial decline, and dysregulated iron metabolism converge to drive ferroptosis, culminating in irreversible structural damage. Future therapeutic paradigms for ARHL may therefore transcend conventional amplification, shifting toward molecular interventions that target the mechanisms of cellular senescence. Pharmacological agents that chelate iron, neutralize lipid peroxides, or bolster antioxidant defenses—including compounds like DFO and CMS121—represent a promising therapeutic frontier for mitigating ARHL by specifically inhibiting ferroptosis.

### 2.2. The Role of Ferroptosis in Noise-Induced Hearing Loss

Exposure to high-intensity or chronic noise environments is a leading cause of SNHL. This exposure inflicts direct mechanical trauma on cochlear structures, particularly the hair cells and spiral ganglion neurons, which culminates in permanent auditory threshold shifts [34,35]. The pathogenesis of noise-induced hearing loss (NIHL) is multifactorial, involving a cascade of interconnected events including excitotoxicity, Ca^2+^ overload, inflammation, and critically, overwhelming oxidative stress driven by ROS [36].

Following acoustic overstimulation, the heightened metabolic activity in hair cells leads to an overproduction of ROS [37]. Preclinical models of noise-induced hearing loss demonstrate that hallmarks of apoptosis and ferroptosis co-exist within cochlear tissues, and the activation of both pathways is strongly correlated with hearing threshold elevation [38]. This cellular damage is accompanied by a surge in oxidative stress markers within the cochlea, directly linking ROS overproduction to the observed pathology. Currently, there are no FDA-approved clinical interventions to effectively prevent or reverse NIHL, highlighting an urgent need for novel therapeutic strategies. While apoptosis, mediated by effectors like Caspase-3, is a known component of NIHL-induced cell death [39], recent attention has shifted towards the role of ferroptosis. Recent evidence links the iron-dependent cell death in NIHL to dysregulated iron metabolism mediated by transferrin receptor 1 (TfR1) [38]. Other signaling pathways, such as the energy-sensing AMP-activated protein kinase (AMPK), are also implicated in the pathogenesis by mediating synaptic loss, further expanding the network of potential targets.

Increasing evidence indicates a strong link between ferroptosis and inflammation, establishing it as a pro-inflammatory cell death pathway. Consequently, many antioxidants that inhibit ferroptosis also exhibit potent anti-inflammatory properties. Central to the endogenous defense against ferroptosis is the enzyme GPX4, which detoxifies phospholipid hydroperoxides (PL-OOH) using GSH as a substrate, thereby preventing lipid peroxidation [40]. The pharmacological inhibitor Ferrostatin-1 (Fer-1) has provided direct evidence for this pathway’s role, demonstrating its ability to attenuate hearing loss in preclinical NIHL models and thus validating ferroptosis as a key pathogenic mechanism. Ferrostatin-1, a specific inhibitor of ferroptosis, has demonstrated significant otoprotective efficacy in preclinical models. Not only does it directly block the lipid peroxidation chain reaction as a free radical scavenging antioxidant, but it also achieves dual inhibition of ferroptosis and apoptosis by downregulating TfR1 expression and suppressing the p53-AIFM2 pathway [38]. In murine models, treatment with Fer-1 has demonstrated significant otoprotective effects, including the attenuation of noise-induced auditory brainstem response threshold shifts and the preservation of OHCs [26]. These findings position the inhibition of regulated hair cell death, specifically ferroptosis, as a promising therapeutic strategy for NIHL. However, the translational journey from preclinical success to clinical application requires rigorous validation of efficacy and safety. Future research efforts should be directed toward developing novel inhibitors with superior pharmacokinetic profiles and establishing targeted local delivery strategies to enhance therapeutic outcomes for NIHL.

### 2.3. The Role of Ferroptosis in Ototoxic Drug-Induced Hearing Loss

Drug-induced ototoxicity is a significant cause of iatrogenic sensorineural hearing loss, with aminoglycoside antibiotics and platinum-based chemotherapeutics like cisplatin being the most implicated agents. Certain aminoglycosides (e.g., gentamicin, tobramycin) predominantly induce vestibular toxicity, whereas others like amikacin, along with cisplatin, primarily cause cochleotoxicity [41]. The underlying mechanism involves not only the disruption of hair cell ion homeostasis but also the induction of excessive ROS production, which activates multiple cell death pathways, including apoptosis, necroptosis, and ferroptosis [42]. Similarly to NIHL, drug-induced ototoxicity typically results in irreversible hearing loss, with damage often beginning in the high-frequency region of the cochlea.

The pathogenesis of cisplatin-induced ototoxicity is strongly linked to oxidative stress, with key molecular drivers including excessive ROS production, depletion of intracellular GSH, and inhibition of antioxidant enzymes within the cochlea [43,44]. This damage exhibits a distinct spatial pattern, with the most severe hair cell loss occurring at the base of the cochlea, corresponding to the region responsible for high-frequency hearing. Cisplatin uptake into hair cells is mediated by transporters like CTR1 and OCTs [45,46]. Intracellularly, while its DNA adducts classically activate p53-mediated apoptosis [47]. Concurrently, emerging evidence implicates ferroptosis, a distinct iron-dependent form of regulated cell death, as a pivotal mechanism in cisplatin-induced ototoxicity [48]. This canonical pathway involves Bax-mediated mitochondrial cytochrome c release and subsequent caspase-3 activation [49,50]. The initiation of ferroptosis is driven by both cisplatin-induced lipid peroxidation and the compromise of the cochlea’s intrinsic antioxidant defenses, most notably the diminished activity of the key enzyme GPX4 [51]. Subsequent studies have established that the downregulation or functional loss of GPX4, the core negative regulator of ferroptosis, is a central event in cisplatin ototoxicity that renders hair cells highly vulnerable to oxidative damage. Targeted deletion of GPX4 in hair cells is sufficient to induce ferroptosis in outer hair cells and cause hearing loss, whereas the loss of FSP1, a parallel ferroptosis inhibitor, has no discernible effect on hearing, underscoring the dominant role of the GPX4 pathway in cochlear homeostasis [48]. This solidifies the role of ferroptosis in drug ototoxicity and highlights therapeutic strategies targeting this pathway.

Intervention strategies targeting the ferroptosis pathway show promising application prospects. For example, the ferroptosis inhibitor Fer-1 protects auditory hair cells by compensating for GPX4 deficiency via its radical-trapping antioxidant activity, thereby mitigating damage induced by both cisplatin and GPX4 deletion [38,48,52]. In HEI-OC1 auditory cells, cisplatin was shown to induce cardinal features of ferroptosis, including lipid peroxidation and labile iron accumulation. Notably, several natural antioxidant compounds, including luteolin, sarsasapogenin, and α-lipoic acid (α-LA), effectively suppressed ferroptosis and protected hair cells by mitigating oxidative stress, upregulating GPX4 expression, or reducing intracellular iron levels [42,48,53]. This highlights the potential of natural products in the development of otoprotective drugs. Similarly, the synthetic inhibitor Fer-1 demonstrated potent protective effects against damage caused by cisplatin and aminoglycosides (e.g., neomycin) in auditory cell lines and cochlear explant models [53]. While these preclinical findings are promising, research into otoprotective ferroptosis inhibitors is still in its early stages. Future preclinical and clinical studies are crucial to validate the safety and efficacy of these inhibitors, paving the way for their potential use in humans.

### 2.4. The Role of Ferroptosis in Sudden Sensorineural Hearing Loss

Sudden sensorineural hearing loss (SSNHL) is an otologic emergency of unknown etiology, clinically defined as a sensorineural hearing loss of at least 30 dB across three or more consecutive frequencies within 72 h. Although viral infections, vascular compromise, and autoimmune disorders are commonly proposed etiologies, approximately 90% of SSNHL cases are ultimately classified as idiopathic [54]. Glucocorticoids represent the current mainstay of treatment for SSNHL [55]. However, the efficacy of this approach is inconsistent, with significant interindividual variability and a substantial proportion of patients achieving incomplete or no hearing recovery. This therapeutic limitation underscores the urgent need to investigate the molecular pathophysiology of SSNHL from novel perspectives.

In recent years, ferroptosis has emerged as a novel focus in SNHL research. As terminally differentiated cells, inner ear hair cells and spiral ganglion neurons are exquisitely sensitive to oxidative stress, a feature that renders them prime targets for ferroptosis. Evidence supporting the role of ferroptosis in SSNHL spans genetic, epigenetic, and therapeutic domains. Genetically, Tisato et al. discovered a significantly higher prevalence of the homozygous-8GG genotype of the iron exporter gene *SLC40A1* in SSNHL patients compared to healthy controls, implicating dysregulated iron metabolism in disease susceptibility. Epigenetically, the same study reported a significant negative correlation between the methylation levels of LINE-1 elements (a surrogate for global DNA methylation) and the severity of hearing loss, suggesting that epigenetic dysregulation contributes to disease progression [56]. From a therapeutic standpoint, research by Bai et al. provides further support: co-administration of N-acetylcysteine (NAC)—a GSH precursor—with intratympanic dexamethasone significantly improved high-frequency hearing recovery in SSNHL patients [57]. This clinical result highlights the therapeutic potential of targeting the antioxidant system to counteract ferroptosis.

Based on the available evidence, we propose a unifying model for ferroptosis in SSNHL. In this model, initiating factors such as aging, genetic susceptibility, and environmental stress disrupt intracellular iron homeostasis (e.g., via impaired FPN1 function) and compromise antioxidant defenses (e.g., via reduced SOD2 activity). These perturbations synergistically induce mitochondrial dysfunction and excessive ROS production, which, in the presence of labile iron, culminates in massive lipid peroxide accumulation and the execution of ferroptosis in auditory cells. This ferroptosis-centric model provides a novel integrative framework for understanding the complex and often idiopathic etiology of SSNHL. In this framework, dysregulated iron metabolism and weakened antioxidant defenses constitute the core vulnerability, while epigenetic modulation and age-related changes act as key contributors to disease onset. Future research should validate key elements of this pathway in larger clinical cohorts and actively explore drugs that directly target ferroptosis.

## 3. Ferroptosis and Metabolic Pathways

In 2012, Dixon coined the term ferroptosis [58] to describe a novel form of regulated cell death driven by iron-dependent lipid peroxidation [59]. Ferroptosis belongs to the broader family of RCD, which also includes well-known pathways like apoptosis and various forms of regulated necrosis (e.g., necroptosis, pyroptosis). All RCD pathways are orchestrated by genetically encoded molecular machinery executing a precise sequence of events [60]. Distinct from apoptosis, ferroptosis is a form of regulated necrosis characterized by an overwhelming loss of cellular reductive capacity. Its execution is uniquely dependent on the dysregulation of cellular redox homeostasis, distinguishing it from other necrotic pathways that often rely on specific signaling complexes [61].

The biochemical hallmarks of ferroptosis are intimately linked: iron accumulation catalyzes the overproduction of ROS, which in turn drives the lethal lipid peroxidation that culminates in plasma membrane rupture [62]. This intricately regulated cell death pathway involves a cascade of molecular events, including glutathione depletion, activation of lipoxygenases, accumulation of reactive oxygen species [63], and disruption of mitochondrial homeostasis. Morphologically, cells undergoing ferroptosis exhibit distinct features that differ from apoptosis and necroptosis. These include shrunken mitochondria with increased membrane density and reduced or vanished cristae, while the nucleus typically remains intact without chromatin condensation [64]. Mitochondria play a dual role in initiating ferroptosis: they are a major source of ROS production (e.g., via the electron transport chain) and are also central hubs for iron metabolism. Ferroptotic stimuli, such as cysteine deprivation, upregulate mitochondrial metabolism (e.g., glutaminolysis), which heightens electron transport chain activity and leads to a burst of ROS [56]. These mitochondrial-derived ROS then oxidize polyunsaturated fatty acids within the mitochondrial membranes, initiating and propagating the lipid peroxidation chain reaction. Mitochondria also function as central hubs for cellular iron metabolism. Labile iron released via ferritinophagy can be taken up by mitochondria, where it catalyzes Fenton reactions and exacerbates ROS-mediated damage [33]. Concurrently, ROS generated from other cellular sources, such as NOX enzymes, can also converge upon the mitochondria. The combination of excessive ROS and ATP depletion can induce the opening of the mitochondrial permeability transition pore (mPTP), leading to mitochondrial swelling and rupture, which potently accelerates the execution of ferroptosis [65,66].

The process of autophagy, a cellular degradation and recycling system, is intricately linked to ferroptosis by regulating intracellular iron availability [67]. Specifically, a selective form of autophagy known as ferritinophagy mediates the degradation of ferritin, the primary iron-storage protein. This process is driven by the cargo receptor NCOA4, which delivers ferritin to the lysosome for breakdown, thereby releasing labile iron into the cytoplasm. Beyond autophagy, the master antioxidant transcription factor, nuclear factor erythroid 2-related factor 2 (NRF2), plays a pivotal protective role against ferroptosis [48]. NRF2 mitigates oxidative stress by transcriptionally activating a suite of antioxidant genes [68]; it also indirectly regulates ferritinophagy and iron homeostasis by modulating key factors such as HERC2 and VAMP8 [69].

The core molecular mechanism of ferroptosis involves the regulation of the balance between oxidative damage and antioxidant defense. The primary process of ferroptosis is the iron-dependent generation of ROS, which causes membrane damage through lipid peroxidation. This process is initiated when the delicate balance is tipped, and ROS generation overwhelms the cellular antioxidant scavenging capacity. This state of oxidative stress then triggers rampant lipid peroxidation and subsequent membrane damage [63]. Several factors act as pro-ferroptotic drivers: ROS, PUFAs, and the iron-catalyzed Fenton reaction collectively execute the lipid peroxidation process. In contrast, a powerful defensive network, centered on the System Xc-GSH-GPX4 axis, counteracts this process by maintaining cellular redox homeostasis and detoxifying lipid peroxides [70]. Thus, ferroptosis can be triggered by disrupting this balance from multiple angles: inhibiting the System Xc^−^ transporter, promoting iron accumulation, or directly inactivating GPX4 In the circulation, iron is transported by transferrin, primarily in its ferric (Fe^3+^) state, and is taken up by cells upon binding to TFR1. The elucidation of ferroptosis not only enriches our understanding of cell death diversity but, crucially for biomedicine, unveils a rich landscape of novel therapeutic targets for treating a wide range of diseases.

**Figure 1:**
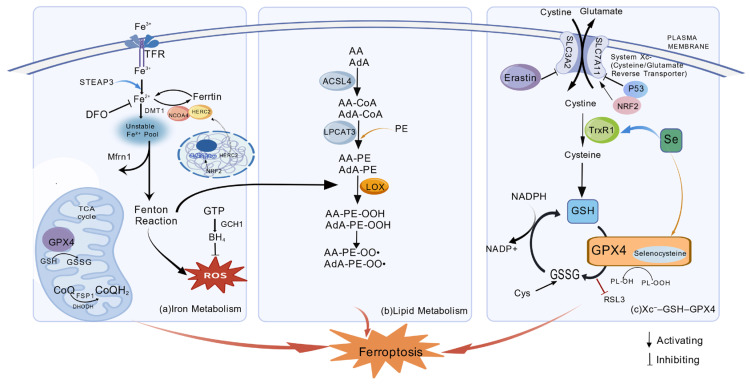
Core molecular pathways regulating ferroptosis. (**a**) Iron Metabolism: Iron uptake and ferritinophagy expand the labile Fe^2+^ pool, which drives reactive oxygen species (ROS) production via the Fenton reaction. (**b**) Lipid Metabolism: Polyunsaturated fatty acids (PUFAs) are incorporated into membrane phospholipids by ACSL4 and LPCAT3, creating vulnerable substrates for peroxidation. (**c**) Antioxidant Defense: The System Xc^−^–GSH–GPX4 axis is the key defense mechanism, where GPX4 uses glutathione (GSH) to neutralize lipid peroxides. This pathway can be blocked by inhibitors like erastin and RSL3, leading to lipid peroxidation and ferroptosis.

The fundamental molecular pathways regulating iron ptosis are shown in Figure 1.

### 3.1. Iron Metabolism Regulates Ferroptosis

As the eponymous metal of ferroptosis, iron is the indispensable catalytic driver of this cell death pathway; its redox activity enables it to drive lipid peroxidation via the Fenton reaction, which forms the core chemical basis of this process. Consequently, the intricate networks governing intracellular iron metabolism—including its uptake, storage, and release—are critical determinants of a cell’s sensitivity to ferroptosis. The intracellular labile iron pool (LIP) is the key mediator of this process, catalyzing the Fenton reaction to convert hydrogen peroxide (H_2_O_2_) into highly reactive hydroxyl radicals (·OH) [25]. These radicals subsequently attack membrane polyunsaturated fatty acids, initiating the self-propagating chain reaction of lipid peroxidation. Beyond the Fenton reaction, iron also serves as a critical cofactor for pro-ferroptotic enzymes, such as lipoxygenases, further underscoring its multifaceted role in executing lipid peroxidation.

In the circulation, iron exists predominantly as ferric iron (Fe^3+^) bound to transferrin. Binding of this complex to the transferrin receptor 1 (TfR1) initiates receptor-mediated endocytosis. Within the acidified endosome, Fe^3+^ is reduced to Fe^2+^ by reductases like STEAP3 and subsequently transported into the cytoplasm by DMT1 [71]. The expression of TfR1 is a critical node in regulating iron uptake; consequently, its upregulation increases iron influx and sensitizes cells to ferroptosis, making elevated TfR1 expression a hallmark of this process [72]. To prevent toxicity, excess intracellular iron is sequestered within ferritin, a nanocage protein that buffers the labile iron pool and maintains iron homeostasis [73,74]. Conversely, ferritin can be degraded via a selective autophagy process termed ferritinophagy. This degradation massively expands the labile iron pool, providing abundant catalytic iron to drive the Fenton reaction and sensitize cells to ferroptosis.

At the cellular level, iron regulatory proteins (IRP1 and IRP2) function as sensors of the labile iron pool, post-transcriptionally regulating iron metabolism genes by binding to iron-responsive elements (IREs) within their mRNAs. Under conditions of iron deficiency, IRPs bind to the 5′ IRE of ferritin mRNA to repress its translation and to the 3′ IRE of TfR1 mRNA to enhance its stability. This coordinated response maximizes iron uptake while minimizing its storage, a state that can sensitize cells to ferroptosis [75,76]. Conversely, under iron-replete conditions, this regulation is reversed to promote iron storage and limit further uptake. At the systemic level, the liver-secreted peptide hormone hepcidin serves as the master regulator of iron homeostasis. Hepcidin controls systemic iron levels by binding to the sole known cellular iron exporter, ferroportin (FPN1), triggering its internalization and degradation. High hepcidin levels thus lead to cellular iron retention and reduced dietary absorption, a state that can increase ferroptotic susceptibility in iron-storing cells [77]. Conversely, low hepcidin levels, as observed in hereditary hemochromatosis, result in uncontrolled iron efflux and systemic iron overload, creating a pro-ferroptotic environment [78].

In summary, iron is not a passive substrate, but rather the central catalyst and a critical regulatory node in ferroptosis. Each step in iron metabolism—from TfR1-mediated uptake and ferritin-based storage to its release via ferritinophagy and its regulation by the IRP/IRE and systemic hepcidin systems—meticulously controls the labile iron pool available for lipid peroxidation. The precise balance of these regulatory networks ultimately dictates a cell’s susceptibility to ferroptosis.

### 3.2. Lipid Metabolism Involved in Ferroptosis

The initiation of ferroptosis is critically dependent on an imbalance between pro-oxidant insults and the cell’s antioxidant defense systems. This imbalance allows for the two fundamental processes to trigger oxidative membrane damage: iron accumulation, which provides the catalyst, and lipid peroxidation, which is the executionary event. Indeed, lipid metabolism plays a central role in determining cellular sensitivity to ferroptosis, particularly through the synthesis and modification of PUFAs.

The abundance of bis-allylic carbons in PUFAs renders them uniquely susceptible to oxidative attack, a property that is central to their role in ferroptosis. Consequently, cellular sensitivity to ferroptosis is largely determined by the efficient activation of these PUFAs and their subsequent incorporation into membrane phospholipids. This two-step process is orchestrated by acyl-CoA synthetase long-chain family member 4 (ACSL4), which activates long-chain PUFAs (preferentially arachidonic and adrenic acid) into PUFA-CoAs, and lysophosphatidylcholine acyltransferase 3 (LPCAT3), which then esterifies these PUFA-CoAs into membrane phospholipids, thereby generating the requisite substrates for lipid peroxidation [79,80]. CRISPR screens have confirmed that ACSL4 is essential for ferroptosis, as it loads PUFAs for their subsequent peroxidation. The pivotal role of ACSL4 is underscored by the profound resistance to ferroptosis observed in cells where it is knocked out. Strikingly, even in the absence of the key defense enzyme GPX4, the concurrent knockout of ACSL4 completely abrogates ferroptosis, demonstrating that without the proper lipid substrate, the cell death program cannot proceed [81,82,83].

The lipid peroxidation that executes ferroptosis can be initiated through two distinct mechanisms: enzymatically via the direct oxidation of PUFA-containing phospholipids (PUFA-PLs) by lipoxygenases (LOXs), or non-enzymatically through iron-catalyzed ROS-driven auto-oxidation [84]. Lipidomic analyses have identified phosphatidylethanolamines (PEs) containing arachidonic acid (AA) or adrenic acid (AdA) as the principal substrates for peroxidation during ferroptosis [85], Accordingly, ACSL4 deficiency specifically depletes these AA/AdA-PE species, thus conferring resistance to RSL3-induced lipid peroxidation. Cells can counteract ferroptosis by remodeling their membrane lipid composition. For instance, the incorporation of monounsaturated fatty acids (MUFAs) via ACSL3 can dilute membrane PUFAs and reduce oxidative susceptibility. Conversely, studies show that ω-6 PUFAs are more potent in promoting ferroptosis than ω-3 PUFAs, indicating that the precise position of double bonds, rather than the degree of unsaturation alone, is a key determinant of a PUFA’s pro-ferroptotic activity [82].

In summary, ACSL4 functions as a critical determinant of ferroptosis by selectively channeling long-chain ω-6 PUFAs into membrane phospholipids, thereby supplying the requisite substrates for lipid peroxidation. This central role establishes ACSL4 as both a key biomarker of cellular susceptibility to ferroptosis and a promising therapeutic target for diseases driven by this cell death pathway.

### 3.3. The Xc^−^–GSH–GPX4 Antioxidant Axis

The canonical pathway defending against ferroptosis is initiated by the glutamate-cystine antiporter, System Xc^−^. This plasma membrane transporter is a heterodimer composed of the light-chain subunit SLC7A11 and the heavy-chain subunit SLC3A2 (4F2hc) [86]. System Xc^−^ mediates the uptake of extracellular cystine in exchange for intracellular glutamate. Once inside the cell, cystine is reduced to cysteine, which then serves as the rate-limiting substrate for the synthesis of the tripeptide antioxidant glutathione (GSH) [86,87]. Glutathione peroxidase 4 (GPX4), a selenocysteine-containing enzyme, then utilizes GSH as a cofactor to detoxify phospholipid hydroperoxides into their corresponding non-toxic alcohols, thereby terminating the lipid peroxidation chain reaction and suppressing ferroptosis. Although GPX4 has cytoplasmic, mitochondrial, and nuclear isoforms, the cytoplasmic form plays the dominant role in preventing ferroptosis. Consequently, pharmacological or genetic disruption of any node in this axis—such as inhibiting System Xc^−^ with erastin or GPX4 with RSL3—leads to the accumulation of lipid peroxides and the execution of ferroptosis [10,88,89,90].

Selenium is indispensable for GPX4 activity, as its synthesis as a selenoprotein requires a specialized mechanism of UGA codon recoding. This process is directed by a SECIS element in the 3′-UTR of GPX4 mRNA, which enables the ribosome to interpret the UGA codon as a signal for selenocysteine incorporation rather than translation termination [22]. Selenium deficiency impairs GPX4 maturation and function, thereby sensitizing cells to ferroptosis [22,91]. Cellular selenium is acquired through two primary routes: the LRP8-mediated endocytosis of liver-derived selenoprotein P, and the System Xc^−^-dependent uptake and reduction of inorganic selenite. In cancer models like MYCN-amplified neuroblastoma, LRP8 deficiency has been shown to induce ferroptosis, highlighting selenium metabolism as a potential anticancer target [52,92,93].

In fact, the pharmacological inhibition of SLC7A11-mediated cystine uptake by compounds such as erastin was the seminal discovery that defined ferroptosis as a distinct form of regulated cell death [58]. This established System Xc^−^ as the primary molecular target of erastin and a prototypical vulnerability for inducing ferroptosis [92]. This discovery underscores the critical importance of System Xc^−^ in maintaining cellular redox homeostasis and protecting against ferroptosis.

### 3.4. Pharmacological Modulation of Ferroptosis

The elucidation of the molecular machinery governing ferroptosis has unveiled a wide array of druggable nodes, enabling the development of compounds that can either induce or inhibit this process. These pharmacological tools are not only crucial for dissecting the pathway’s intricacies, but also hold immense promise for therapeutic interventions in diseases like SNHL.

#### 3.4.1. Inducers of Ferroptosis as Research Probes

A class of compounds known as ferroptosis inducers have been instrumental in defining the pathway’s core components. These are generally categorized based on their mechanism of action. Class I inducers, exemplified by erastin and sulfasalazine, function by inhibiting the System Xc^−^ transporter, thereby depleting cellular GSH pools [94]. Class II inducers, such as RSL3, act more directly by covalently binding to and inactivating the central defense enzyme, GPX4 [95,96]. A third type of inducer, FIN56, promotes ferroptosis through a distinct mechanism involving the degradation of GPX4 protein and the activation of squalene synthase [97]. While these inducers are primarily used as experimental tools, their mechanisms of action have provided a clear roadmap for designing protective strategies.

#### 3.4.2. Inhibitors of Ferroptosis as Therapeutic Agents

More relevant to the treatment of SNHL are the ferroptosis inhibitors, which counteract the cell death process by targeting different key steps within the pathway. The most well-characterized class of inhibitors are the radical-trapping antioxidants (RTAs). Prototypical examples like Fer-1 and Liproxstatin-1 are potent lipophilic antioxidants that act downstream of GPX4 [38,58,98]. Instead of restoring GPX4 function, they directly scavenge lipid peroxyl radicals, thereby breaking the chain reaction of lipid peroxidation. As previously discussed, Fer-1 has shown significant otoprotective effects in preclinical models of both noise-induced and drug-induced hearing loss.

Another direct and effective inhibitory strategy is the use of iron chelators. Since iron is the indispensable catalyst for ferroptosis, sequestering excess labile iron can halt the process at its origin. The clinically approved iron chelator, Deferoxamine (DFO), has been shown to protect auditory cells from oxidative stress and ferroptosis by binding free iron and preventing its participation in the Fenton reaction [98]. This approach validates iron overload as one of therapeutic target in SNHL.

Highlighting the potential of naturally driven biomedicines, several natural products have also been identified as potent ferroptosis inhibitors. For example, flavonoids like quercetin exhibit otoprotective effects, likely through combined iron-chelation and antioxidant activities, while epigallocatechin gallate bolsters cellular defenses via NRF2 activation [2,99]. Other compounds act on iron metabolism: the alkaloid nuciferine inhibits NCOA4-mediated ferritinophagy, thereby restricting labile iron supply, while curcumin exerts pleiotropic effects by regulating iron-related proteins and lipid peroxidation [48,100]. A broad spectrum of other natural products also exhibit anti-ferroptotic properties, including resveratrol [79], alpha-lipoic acid [101], ginkgo biloba extract [102], tanshinone IIA [8], tetramethylpyrazine [103], berberine [104], and ginsenosides [105], which act through diverse mechanisms such as inhibiting iron accumulation, suppressing lipid peroxidation, or modulating multiple protective pathways.

To enhance targeting and bioavailability, various nanocarriers have been developed for antioxidant delivery. For example, loading ferrostatin-1 into nanoparticles facilitates its targeted accumulation within the cochlea [31]. At the subcellular level, nanocarriers can enable the precise delivery of mitochondria-targeted antioxidants, such as MitoQ and MitoTEMPO, thereby blocking ferroptosis at its site of initiation [77]. Furthermore, nanocarrier systems, including liposomes and polymeric nanoparticles, are employed to enhance the otherwise poor solubility and stability of natural products such as quercetin and curcumin [79]. Recent discoveries have also revealed GPX4-independent mechanisms for suppressing ferroptosis. For instance, the ferroptosis suppressor protein 1 (FSP1), also known as AIFM2, can reduce coenzyme Q10 to its antioxidant form, which then traps lipid radicals in parallel to the GSH/GPX4 system [106,107]. The non-canonical vitamin K cycle has been identified as another potent, parallel ferroptosis suppression pathway [108]. Modulating these alternative pathways could offer new therapeutic avenues, especially in conditions where the canonical GPX4 axis is compromised.

## 4. Conclusions and Future Perspectives

While ferroptosis is increasingly implicated as a pathogenic mechanism in diverse forms of hearing loss, its precise contribution to the onset and progression of each etiology remains to be fully elucidated. Its precise role in the onset and progression of different hearing loss conditions requires further confirmation. Among multiple contributing factors such as aging, noise exposure, and ototoxic injury, iron metabolism disorders and the resulting lipid peroxidation have been observed as a potential common pathway driving damage to cochlear structures. This insight not only offers a new perspective for understanding the pathogenesis of sensorineural hearing loss, but also suggests potential therapeutic targets.

Pharmacological strategies aimed at inhibiting ferroptosis—such as iron chelation, the use of radical-trapping antioxidants like Ferrostatin-1, and leveraging natural bioactive compounds—have shown significant otoprotective potential in preclinical studies. These findings strongly support the modulation of ferroptosis as a viable therapeutic strategy for preserving hearing.

Despite these promising advances, the translation of these findings into clinical practice faces significant hurdles. Future research must prioritize the development of specific ferroptosis inhibitors with favorable safety profiles and effective inner-ear delivery systems. Identifying reliable biomarkers to monitor ferroptotic activity in patients is another critical step for successful clinical trials. Furthermore, understanding the interplay between ferroptosis and other cell death pathways may unlock opportunities for more effective combination therapies. Overcoming these challenges will be crucial in harnessing our knowledge of ferroptosis to develop novel and effective treatments for the millions affected by sensorineural hearing loss.

## Data Availability

No new data were created or analyzed in this study. Data sharing is not applicable to this article.

## Abbreviations

The following abbreviations are used in this manuscript:

ACSL4	Acyl-CoA synthetase long-chain family member 4
ARHL	Age-related hearing loss
ATP	Adenosine triphosphate
DMT1	Divalent metal transporter 1
Fe^2+^	Ferrous ion
Fe^3+^	Ferric ion
Fer-1	Ferrostatin-1
GPX4	Glutathione peroxidase 4
GSH	Glutathione
LOX	Lipoxygenase
NIHL	Noise-induced hearing loss
ODIHL	Ototoxic drug-induced hearing loss
OHCs	Outer hair cells
PUFAs	Polyunsaturated fatty acids
RCD	Regulated cell death
ROS	Reactive oxygen species
RSL3	RAS-selective lethal 3
SLC7A11	Solute carrier family 7 member 11
SNHL	Sensorineural hearing loss
STEAP3	Six-transmembrane epithelial antigen of the prostate 3
TFR	Transferrin receptor
MUFA	Monounsaturated fatty acids
HMG	β-Hydroxy-β-methylglutaric acid (HMG)
NCOA4	Nuclear Coactivator 4
TrxR1	Thioredoxin Reductase 1
MFRN1	Mitoferrin-1
DHODH	Dihydroorotate dehydrogenase

## References

[1] Chadha S., Kamenov K., Cieza A. (2021). The world report on hearing, 2021. Bull. World Health Organ..

[2] Kim E., Han S.Y., Hwang K., Kim D., Kim E.M., Hossain M.A., Kim J.H., Cho J.Y. (2019). Antioxidant and cytoprotective effects of (-)-epigallocatechin-3-(3″-O-methyl) gallate. Int. J. Mol. Sci..

[3] Kujawa S.G., Liberman M.C. (2009). Adding insult to injury: Cochlear nerve degeneration after “temporary” noise-induced hearing loss. J. Neurosci..

[4] Fetoni A.R., De Bartolo P., Eramo S.L., Rolesi R., Paciello F., Bergamini C., Fato R., Paludetti G., Petrosini L., Troiani D. (2013). Noise-induced hearing loss (NIHL) as a target of oxidative stress-mediated damage: Cochlear and cortical responses after an increase in antioxidant defense. J. Neurosci..

[5] Shi T.F., Zhou Z., Jiang W.J., Huang T.L., Si J.Q., Li L. (2024). Hyperglycemia-induced oxidative stress exacerbates mitochondrial apoptosis damage to cochlear stria vascularis pericytes via the ROS-mediated Bcl-2/CytC/AIF pathway. Redox Rep..

[6] Yu F., Zhang Q., Liu H., Liu J., Yang S., Luo X., Liu W., Zheng H., Liu Q., Cui Y. (2022). Dynamic O-GlcNAcylation coordinates ferritinophagy and mitophagy to activate ferroptosis. Cell Discov..

[7] Zheng Z., Zhang S., Liu X., Wang X., Xue C., Wu X., Zhang X., Xu X., Liu Z., Yao L. (2024). LRRK2 regulates ferroptosis through the system Xc-GSH-GPX4 pathway in the neuroinflammatory mechanism of Parkinson’s disease. J. Cell. Physiol..

[8] Gong C., Fu X., Ma Q., He M., Zhu X., Liu L., Zhou D., Yan S. (2025). Gastrodin: Modulating the xCT/GPX4 and ACSL4/LPCAT3 pathways to inhibit ferroptosis after ischemic stroke. Phytomedicine.

[9] Kraft V.A.N., Bezjian C.T., Pfeiffer S., Ringelstetter L., Müller C., Zandkarimi F., Merl-Pham J., Bao X., Anastasov N., Kössl J. (2020). GTP Cyclohydrolase 1/Tetrahydrobiopterin counteract ferroptosis through lipid remodeling. ACS Cent. Sci..

[10] Liu N., Wu W.L., Wan X.R., Wang J., Huang J.N., Jiang Y.Y., Sheng Y.C., Wu J.C., Liang Z.Q., Qin Z.H. (2024). Regulation of FSP1 myristoylation by NADPH: A novel mechanism for ferroptosis inhibition. Redox Biol..

[11] Nakamura T., Hipp C., Santos Dias Mourão A., Borggräfe J., Aldrovandi M., Henkelmann B., Wanninger J., Mishima E., Lytton E., Emler D. (2023). Phase separation of FSP1 promotes ferroptosis. Nature.

[12] Bi G., Liang J., Bian Y., Shan G., Huang Y., Lu T., Zhang H., Jin X., Chen Z., Zhao M. (2024). Polyamine-mediated ferroptosis amplification acts as a targetable vulnerability in cancer. Nat. Commun..

[13] Wang Z.L., Yuan L., Li W., Li J.Y. (2022). Ferroptosis in Parkinson’s disease: Glia-neuron crosstalk. Trends Mol. Med..

[14] Jinson S., Zhang Z., Lancaster G.I., Murphy A.J., Morgan P.K. (2025). Iron, lipid peroxidation, and ferroptosis play pathogenic roles in atherosclerosis. Cardiovasc. Res..

[15] Wang P., Cui Y., Ren Q., Yan B., Zhao Y., Yu P., Gao G., Shi H., Chang S., Chang Y.Z. (2021). Mitochondrial ferritin attenuates cerebral ischaemia/reperfusion injury by inhibiting ferroptosis. Cell Death Dis..

[16] Zheng J., Shen W., He D.Z., Long K.B., Madison L.D., Dallos P. (2000). Prestin is the motor protein of cochlear outer hair cells. Nature.

[17] Fettiplace R., Hackney C.M. (2006). The sensory and motor roles of auditory hair cells. Nat. Rev. Neurosci..

[18] Liberman M.C., Gao J., He D.Z., Wu X., Jia S., Zuo J. (2002). Prestin is required for electromotility of the outer hair cell and for the cochlear amplifier. Nature.

[19] Marcotti W. (2012). Functional assembly of mammalian cochlear hair cells. Exp. Physiol..

[20] Keithley E.M. (2022). Inner ear immunity. Hear. Res..

[21] Chen F.Q., Zheng H.W., Hill K., Sha S.H. (2012). Traumatic noise activates Rho-family GTPases through transient cellular energy depletion. J. Neurosci..

[22] Yao Y., Chen Z., Zhang H., Chen C., Zeng M., Yunis J., Wei Y., Wan Y., Wang N., Zhou M. (2021). Selenium-GPX4 axis protects follicular helper T cells from ferroptosis. Nat. Immunol..

[23] Rance G., Chisari D. (2016). Auditory neuropathy in a patient with hemochromatosis. J. Otol..

[24] Warnke C., Andersen K., Hartung H.P., Hefter H. (2011). Superficial siderosis of the central nervous system in a patient with hemochromatosis and Wilson’s disease. Dtsch. Med. Wochenschr..

[25] Meng S., Zhou P., Sun Y., Zhang P., Zhou C., Xiong Z., Zhang H., Liang J., Lai B. (2022). Reducing agents enhanced Fenton-like oxidation (Fe(III)/Peroxydisulfate): Substrate specific reactivity of reactive oxygen species. Water Res..

[26] Lyu A.R., Kim T.H., Park S.J., Shin S.A., Jeong S.H., Yu Y., Huh Y.H., Je A.R., Park M.J., Park Y.H. (2020). Mitochondrial damage and necroptosis in aging cochlea. Int. J. Mol. Sci..

[27] Xu Y., Yang W.P., Hu B.H., Yang S., Henderson D. (2017). Involvement of p53 and Bcl-2 in sensory cell degeneration in aging rat cochleae. Acta Otolaryngol..

[28] Wu P.Z., O’Malley J.T., de Gruttola V., Liberman M.C. (2020). Age-related hearing loss is dominated by damage to inner ear sensory cells, not the cellular battery that powers them. J. Neurosci..

[29] Gupta S., Eavey R.D., Wang M., Curhan S.G., Curhan G.C. (2019). Type 2 diabetes and the risk of incident hearing loss. Diabetologia.

[30] Someya S., Xu J., Kondo K., Ding D., Salvi R.J., Yamasoba T., Rabinovitch P.S., Weindruch R., Leeuwenburgh C., Tanokura M. (2009). Age-related hearing loss in C57BL/6J mice is mediated by Bak-dependent mitochondrial apoptosis. Proc. Natl. Acad. Sci. USA.

[31] Hu B., Liu Y., Chen X., Zhao J., Han J., Dong H., Zheng Q., Nie G. (2020). Ferrostatin-1 protects auditory hair cells from cisplatin-induced ototoxicity in vitro and in vivo. Biochem. Biophys. Res. Commun..

[32] Pham T.B., Boussaty E.C., Currais A., Maher P., Schubert D.R., Manor U., Friedman R.A. (2023). Attenuation of age-related hearing impairment in Senescence-Accelerated Mouse Prone 8 (SAMP8) mice treated with Fatty Acid Synthase Inhibitor CMS121. J. Mol. Neurosci..

[33] Fuhrmann D.C., Mondorf A., Beifuß J., Jung M., Brüne B. (2020). Hypoxia inhibits ferritinophagy, increases mitochondrial ferritin, and protects from ferroptosis. Redox Biol..

[34] Kurabi A., Keithley E.M., Housley G.D., Ryan A.F., Wong A.C. (2017). Cellular mechanisms of noise-induced hearing loss. Hear. Res..

[35] Wu P.Z., O’Malley J.T., de Gruttola V., Liberman M.C. (2021). Primary neural degeneration in noise-exposed human cochleas: Correlations with outer hair cell loss and word-discrimination scores. J. Neurosci..

[36] Orrenius S., Zhivotovsky B., Nicotera P. (2003). Regulation of cell death: The calcium-apoptosis link. Nat. Rev. Mol. Cell Biol..

[37] Yamane H., Nakai Y., Takayama M., Iguchi H., Nakagawa T., Kojima A. (1995). Appearance of free radicals in the guinea pig inner ear after noise-induced acoustic trauma. Eur. Arch. Otorhinolaryngol..

[38] Ma P.W., Wang W.L., Chen J.W., Yuan H., Lu P.H., Gao W., Ding X.R., Lun Y.Q., Liang R., He Z.H. (2022). Treatment with the ferroptosis inhibitor ferrostatin-1 attenuates noise-induced hearing loss by suppressing ferroptosis and apoptosis. Oxid. Med. Cell. Longev..

[39] Nicotera T.M., Hu B.H., Henderson D. (2003). The caspase pathway in noise-induced apoptosis of the chinchilla cochlea. J. Assoc. Res. Otolaryngol..

[40] Maiorino M., Conrad M., Ursini F. (2018). GPx4, lipid peroxidation, and cell death: Discoveries, rediscoveries, and open issues. Antioxid. Redox Signal..

[41] Leis J.A., Rutka J.A., Gold W.L. (2015). Aminoglycoside-induced ototoxicity. CMAJ.

[42] Chen Z., Xie T., Chen C., Lin T., Wu X., Chen Y., Lin Y., Luo X., Zeng C., Lin C. (2025). Sarsasapogenin protects hair cells from cisplatin-induced ototoxicity by attenuating apoptosis and ferroptosis via alleviating oxidative stress. Front. Pharmacol..

[43] Kopke R.D., Liu W., Gabaizadeh R., Jacono A., Feghali J., Spray D., Garcia P., Steinman H., Malgrange B., Ruben R.J. (1997). Use of organotypic cultures of Corti’s organ to study the protective effects of antioxidant molecules on cisplatin-induced damage of auditory hair cells. Am. J. Otol..

[44] Ramkumar V., Mukherjea D., Dhukhwa A., Rybak L.P. (2021). Oxidative stress and inflammation caused by cisplatin ototoxicity. Antioxidants.

[45] Das A., Ash D., Fouda A.Y., Sudhahar V., Kim Y.M., Hou Y., Hudson F.Z., Stansfield B.K., Caldwell R.B., McMenamin M. (2022). Cysteine oxidation of copper transporter CTR1 drives VEGFR2 signalling and angiogenesis. Nat. Cell Biol..

[46] Maier J., Niello M., Rudin D., Daws L.C., Sitte H.H. (2021). The interaction of organic cation transporters 1-3 and PMAT with psychoactive substances. Handb. Exp. Pharmacol..

[47] Masuda Y., Futamura M., Kamino H., Nakamura Y., Kitamura N., Ohnishi S., Miyamoto Y., Ichikawa H., Ohta T., Ohki M. (2006). The potential role of DFNA5, a hearing impairment gene, in p53-mediated cellular response to DNA damage. J. Hum. Genet..

[48] Gao X., Mao H., Zhao L., Li X., Liao Y., Li W., Li H., Chen Y. (2024). Nuciferine protects cochlear hair cells from ferroptosis through inhibiting NCOA4-mediated ferritinophagy. Antioxidants.

[49] Hazlitt R.A., Min J., Zuo J. (2018). Progress in the development of preventative drugs for cisplatin-induced hearing loss. J. Med. Chem..

[50] Takahashi K., Kamiya K., Urase K., Suga M., Takizawa T., Mori H., Yoshikawa Y., Ichimura K., Kuida K., Momoi T. (2001). Caspase-3-deficiency induces hyperplasia of supporting cells and degeneration of sensory cells resulting in the hearing loss. Brain Res..

[51] Wang X., Zhou Y., Wang D., Wang Y., Zhou Z., Ma X., Liu X., Dong Y. (2023). Cisplatin-induced ototoxicity: From signaling network to therapeutic targets. Biomed. Pharmacother..

[52] Hu K., Li K., Lv J., Feng J., Chen J., Wu H., Cheng F., Jiang W., Wang J., Pei H. (2020). Suppression of the SLC7A11/glutathione axis causes synthetic lethality in KRAS-mutant lung adenocarcinoma. J. Clin. Investig..

[53] Cho S., Hong S.J., Kang S.H., Park Y., Kim S.K. (2022). Alpha-lipoic acid attenuates apoptosis and ferroptosis in cisplatin-induced ototoxicity via the reduction of intracellular lipid droplets. Int. J. Mol. Sci..

[54] Schreiber B.E., Agrup C., Haskard D.O., Luxon L.M. (2010). Sudden sensorineural hearing loss. Lancet.

[55] Newsted D., Bajin M.D., You P. (2025). Sudden sensorineural hearing loss. CMAJ.

[56] Merkel M., Goebel B., Boll M., Adhikari A., Maurer V., Steinhilber D., Culmsee C. (2023). Mitochondrial reactive oxygen species formation determines ACSL4/LPCAT2-mediated ferroptosis. Antioxidants.

[57] Bai X., Chen S., Xu K., Jin Y., Niu X., Xie L., Qiu Y., Liu X.Z., Sun Y. (2021). N-acetylcysteine combined with dexamethasone treatment improves sudden sensorineural hearing loss and attenuates hair cell death caused by ROS stress. Front. Cell Dev. Biol..

[58] Dixon S.J., Lemberg K.M., Lamprecht M.R., Skouta R., Zaitsev E.M., Gleason C.E., Patel D.N., Bauer A.J., Cantley A.M., Yang W.S. (2012). Ferroptosis: An iron-dependent form of nonapoptotic cell death. Cell.

[59] Jiang X., Stockwell B.R., Conrad M. (2021). Ferroptosis: Mechanisms, biology and role in disease. Nat. Rev. Mol. Cell Biol..

[60] Berndt C., Alborzinia H., Amen V.S., Ayton S., Barayeu U., Bartelt A., Bayir H., Bebber C.M., Birsoy K., Böttcher J.P. (2024). Ferroptosis in health and disease. Redox Biol..

[61] Yuan J., Ofengeim D. (2024). A guide to cell death pathways. Nat. Rev. Mol. Cell Biol..

[62] Liang D., Minikes A.M., Jiang X. (2022). Ferroptosis at the intersection of lipid metabolism and cellular signaling. Mol. Cell.

[63] Park E., Chung S.W. (2019). ROS-mediated autophagy increases intracellular iron levels and ferroptosis by ferritin and transferrin receptor regulation. Cell Death Dis..

[64] Krainz T., Gaschler M.M., Lim C., Sacher J.R., Stockwell B.R., Wipf P. (2016). A mitochondrial-targeted nitroxide is a potent inhibitor of ferroptosis. ACS Cent. Sci..

[65] Huang F., Pang J., Xu L., Niu W., Zhang Y., Li S., Li X. (2022). Hedyotis diffusa injection induces ferroptosis via the Bax/Bcl2/VDAC2/3 axis in lung adenocarcinoma. Phytomedicine.

[66] Yang Y., Luo M., Zhang K., Zhang J., Gao T., Connell D.O., Yao F., Mu C., Cai B., Shang Y. (2020). Nedd4 ubiquitylates VDAC2/3 to suppress erastin-induced ferroptosis in melanoma. Nat. Commun..

[67] Glick D., Barth S., Macleod K.F. (2010). Autophagy: Cellular and molecular mechanisms. J. Pathol..

[68] Kajarabille N., Latunde-Dada G.O. (2019). Programmed cell-death by ferroptosis: Antioxidants as mitigators. Int. J. Mol. Sci..

[69] Anandhan A., Dodson M., Shakya A., Chen J., Liu P., Wei Y., Tan H., Wang Q., Jiang Z., Yang K. (2023). NRF2 controls iron homeostasis and ferroptosis through HERC2 and VAMP8. Sci. Adv..

[70] Amaral E.P., Foreman T.W., Namasivayam S., Hilligan K.L., Kauffman K.D., Barbosa Bomfim C.C., Costa D.L., Barreto-Duarte B., Gurgel-Rocha C., Santana M.F. (2022). GPX4 regulates cellular necrosis and host resistance in Mycobacterium tuberculosis infection. J. Exp. Med..

[71] Touret N., Martin-Orozco N., Paroutis P., Furuya W., Lam-Yuk-Tseung S., Forbes J., Gros P., Grinstein S. (2004). Molecular and cellular mechanisms underlying iron transport deficiency in microcytic anemia. Blood.

[72] Feng H., Schorpp K., Jin J., Yozwiak C.E., Hoffstrom B.G., Decker A.M., Rajbhandari P., Stokes M.E., Bender H.G., Csuka J.M. (2020). Transferrin receptor is a specific ferroptosis marker. Cell Rep..

[73] Theil E.C. (2013). Ferritin: The protein nanocage and iron biomineral in health and in disease. Inorg. Chem..

[74] Yu Y., Jiang L., Wang H., Shen Z., Cheng Q., Zhang P., Wang J., Wu Q., Fang X., Duan L. (2020). Hepatic transferrin plays a role in systemic iron homeostasis and liver ferroptosis. Blood.

[75] Wilkinson N., Pantopoulos K. (2014). The IRP/IRE system in vivo: Insights from mouse models. Front. Pharmacol..

[76] Pantopoulos K. (2004). Iron metabolism and the IRE/IRP regulatory system: An update. Ann. N. Y. Acad. Sci..

[77] Liu B., Chen L., Gao M., Dai M., Zheng Y., Qu L., Zhang J., Gong G. (2024). A comparative study of the efficiency of mitochondria-targeted antioxidants MitoTEMPO and SKQ1 under oxidative stress. Free Radic. Biol. Med..

[78] Allen K.J., Gurrin L.C., Constantine C.C., Osborne N.J., Delatycki M.B., Nicoll A.J., McLaren C.E., Bahlo M., Nisselle A.E., Vulpe C.D. (2008). Iron-overload-related disease in HFE hereditary hemochromatosis. N. Engl. J. Med..

[79] Rodrigo R., Retamal C., Schupper D., Vergara-Hernández D., Saha S., Profumo E., Buttari B., Saso L. (2022). Antioxidant cardioprotection against reperfusion injury: Potential therapeutic roles of resveratrol and quercetin. Molecules.

[80] Dixon S.J., Winter G.E., Musavi L.S., Lee E.D., Snijder B., Rebsamen M., Superti-Furga G., Stockwell B.R. (2015). Human haploid cell genetics reveals roles for lipid metabolism genes in nonapoptotic cell death. ACS Chem. Biol..

[81] Soupene E., Fyrst H., Kuypers F.A. (2008). Mammalian acyl-CoA: Lysophosphatidylcholine acyltransferase enzymes. Proc. Natl. Acad. Sci. USA.

[82] Doll S., Proneth B., Tyurina Y.Y., Panzilius E., Kobayashi S., Ingold I., Irmler M., Beckers J., Aichler M., Walch A. (2017). ACSL4 dictates ferroptosis sensitivity by shaping cellular lipid composition. Nat. Chem. Biol..

[83] Huang W., Zhang Y., Das N.K., Solanki S., Jain C., El-Derany M.O., Koo I., Bell H.N., Aabed N., Singhal R. (2025). Fibroblast lipid metabolism through ACSL4 regulates epithelial sensitivity to ferroptosis in IBD. Nat. Metab..

[84] Yin H., Xu L., Porter N.A. (2011). Free radical lipid peroxidation: Mechanisms and analysis. Chem. Rev..

[85] Kagan V.E., Mao G., Qu F., Angeli J.P., Doll S., Croix C.S., Dar H.H., Liu B., Tyurin V.A., Ritov V.B. (2017). Oxidized arachidonic and adrenic PEs navigate cells to ferroptosis. Nat. Chem. Biol..

[86] Sato H., Tamba M., Ishii T., Bannai S. (1999). Cloning and expression of a plasma membrane cystine/glutamate exchange transporter composed of two distinct proteins. J. Biol. Chem..

[87] Stipanuk M.H. (2004). Sulfur amino acid metabolism: Pathways for production and removal of homocysteine and cysteine. Annu. Rev. Nutr..

[88] Dolma S., Lessnick S.L., Hahn W.C., Stockwell B.R. (2003). Identification of genotype-selective antitumor agents using synthetic lethal chemical screening in engineered human tumor cells. Cancer Cell.

[89] Mandal P.K., Seiler A., Perisic T., Kölle P., Banjac Canak A., Förster H., Weiss N., Kremmer E., Lieberman M.W., Bannai S. (2010). System x(c)- and thioredoxin reductase 1 cooperatively rescue glutathione deficiency. J. Biol. Chem..

[90] Stockwell B.R., Friedmann Angeli J.P., Bayir H., Bush A.I., Conrad M., Dixon S.J., Fulda S., Gascón S., Hatzios S.K., Kagan V.E. (2017). Ferroptosis: A regulated cell death nexus linking metabolism, redox biology, and disease. Cell.

[91] Ingold I., Berndt C., Schmitt S., Doll S., Poschmann G., Buday K., Roveri A., Peng X., Porto Freitas F., Seibt T. (2018). Selenium utilization by GPX4 is required to prevent hydroperoxide-induced ferroptosis. Cell.

[92] Koppula P., Zhuang L., Gan B. (2021). Cystine transporter SLC7A11/xCT in cancer: Ferroptosis, nutrient dependency, and cancer therapy. Protein Cell.

[93] Koppula P., Zhang Y., Shi J., Li W., Gan B. (2017). The glutamate/cystine antiporter SLC7A11/xCT enhances cancer cell dependency on glucose by exporting glutamate. J. Biol. Chem..

[94] Hensley C.T., Wasti A.T., DeBerardinis R.J. (2013). Glutamine and cancer: Cell biology, physiology, and clinical opportunities. J. Clin. Investig..

[95] Feng Q., Yang Y., Ren K., Qiao Y., Sun Z., Pan S., Liu F., Liu Y., Huo J., Liu D. (2023). Broadening horizons: The multifaceted functions of ferroptosis in kidney diseases. Int. J. Biol. Sci..

[96] Cui Y., Zhang Z., Zhou X., Zhao Z., Zhao R., Xu X., Kong X., Ren J., Yao X., Wen Q. (2021). Microglia and macrophage exhibit attenuated inflammatory response and ferroptosis resistance after RSL3 stimulation via increasing Nrf2 expression. J. Neuroinflammation.

[97] Sun Y., Berleth N., Wu W., Schlütermann D., Deitersen J., Stuhldreier F., Berning L., Friedrich A., Akgün S., Mendiburo M.J. (2021). Fin56-induced ferroptosis is supported by autophagy-mediated GPX4 degradation and functions synergistically with mTOR inhibition to kill bladder cancer cells. Cell Death Dis..

[98] Lu P.H., Ma P.W., Wang W.L., Gao W., Chen J.W., Yuan H., Ding X.R., Lun Y.Q., Liang R., Li S.Y. (2024). Deferoxamine protects cochlear hair cells and hair cell-like HEI-OC1 cells against tert-butyl hydroperoxide-induced ototoxicity.. Biochim. Biophys. Acta, Mol. Basis Dis..

[99] Liu Z., Zhang H., Hong G., Bi X., Hu J., Zhang T., An Y., Guo N., Dong F., Xiao Y. (2024). Inhibition of Gpx4-mediated ferroptosis alleviates cisplatin-induced hearing loss in C57BL/6 mice. Mol. Ther..

[100] Dehzad M.J., Ghalandari H., Nouri M., Askarpour M. (2023). Antioxidant and anti-inflammatory effects of curcumin/turmeric supplementation in adults: A GRADE-assessed systematic review and dose-response meta-analysis of randomized controlled trials. Cytokine.

[101] Skibska B., Kochan E., Stanczak A., Lipert A., Skibska A. (2023). Antioxidant and anti-inflammatory effects of α-lipoic acid on lipopolysaccharide-induced oxidative stress in rat kidney. Arch. Immunol. Ther. Exp..

[102] Singh S.K., Srivastav S., Castellani R.J., Plascencia-Villa G., Perry G. (2019). Neuroprotective and antioxidant effect of Ginkgo biloba extract against AD and other neurological disorders. Neurotherapeutics.

[103] Li G., Xiao H., Zuo C., Xie H., Wang X., Wang J., Liu Y., Hou Q., Sun G., Tian Y. (2025). N-butylphthalide (NBP) and ligustrazine (TMP) triazole hybrids target the KEAP1-NRF2 pathway to inhibit ferroptosis and exert brain neuroprotectivity. Redox Biol..

[104] Wang T.T., Yu L.L., Zheng J.M., Han X.Y., Jin B.Y., Hua C.J., Chen Y.S., Shang S.S., Liang Y.Z., Wang J.R. (2024). Berberine inhibits ferroptosis and stabilizes atherosclerotic plaque through NRF2/SLC7A11/GPX4 pathway. Chin. J. Integr. Med..

[105] Wu Y., Pi D., Zhou S., Yi Z., Dong Y., Wang W., Ye H., Chen Y., Zuo Q., Ouyang M. (2023). Ginsenoside Rh3 induces pyroptosis and ferroptosis through the Stat3/p53/NRF2 axis in colorectal cancer cells. Acta Biochim. Biophys. Sin..

[106] Bersuker K., Hendricks J.M., Li Z., Magtanong L., Ford B., Tang P.H., Roberts M.A., Tong B., Maimone T.J., Zoncu R. (2019). The CoQ oxidoreductase FSP1 acts parallel to GPX4 to inhibit ferroptosis. Nature.

[107] Doll S., Freitas F.P., Shah R., Aldrovandi M., da Silva M.C., Ingold I., Goya Grocin A., Xavier da Silva T.N., Panzilius E., Scheel C.H. (2019). FSP1 is a glutathione-independent ferroptosis suppressor. Nature.

[108] Mishima E., Ito J., Wu Z., Nakamura T., Wahida A., Doll S., Tonnus W., Nepachalovich P., Eggenhofer E., Aldrovandi M. (2022). A non-canonical vitamin K cycle is a potent ferroptosis suppressor. Nature.

